# Oncolytic virus therapy in hepatocellular carcinoma

**DOI:** 10.32604/or.2025.061857

**Published:** 2025-06-26

**Authors:** YUYU YE, YING LIU

**Affiliations:** Department of Infectious Diseases, The Third Affiliated Hospital of Sun Yat-sen University, Guangzhou, 510630, China

**Keywords:** Oncolytic Virus, Hepatocellular Carcinoma, Oncolytic Virotherapy

## Abstract

Liver cancer is the fifth most common cancer in the world, with China bearing a disproportionate burden of cases. Typically diagnosed at advanced stages, liver cancer often utilizes surgical treatments such as resection, transcatheter hepatic artery chemoembolization (TACE), and radiofrequency ablation. However, advancements in genetic engineering and tumor immunology have unveiled the distinct potential of targeted oncolytic virus therapy. Oncolytic virus, in particular, can selectively destroy tumor cells without harming normal cells, offering a promising avenue for liver cancer treatment through immune system activation, tumor microenvironment modulation, and other mechanisms. This review describes the mechanism of action of oncolytic viruses, the new development of several common oncolytic viruses, and the combination with traditional therapies, aiming to provide directions for the subsequent therapeutic research on hepatocellular carcinoma (HCC).

## Introduction

Liver cancer is the fifth most common cancer and the fourth leading cause of cancer-related deaths worldwide [1], which is a heavy disease burden globally. Major known risk factors associated with hepatocellular carcinoma (HCC) are viral infections (mostly chronic hepatitis B and C), metabolic factors (mostly non-alcoholic fatty liver disease), toxic agents (alcohol and aflatoxins), and immune system-related disorders [2]. Among these, hepatitis B virus infection is the most significant risk factor for the occurrence of liver cancer. HCC accounts for around 90% of primary liver cancer [3], with a higher prevalence in men than in women. Due to the asymptomatic early stage of HCC, a majority of cases are not diagnosed until the disease has progressed to advanced stages, complicating therapeutic intervention. In addressing HCC, it is imperative to take into account not only the dimensions and spread of the tumor but also the severity of the patient’s liver function, given that the majority of therapeutic interventions are likely to exacerbate the pre-existing hepatic impairment. According to the latest guidelines for the diagnosis and treatment of primary liver cancer, the current first-line treatment methods include surgical resection, liver transplantation, ablation therapy, transarterial interventional therapy [4], and systemic antitumor treatment such as molecular targeted therapy, immune checkpoint inhibitor therapy, and others. Although there are a number of therapeutic approaches to treat early and advanced HCC, patients with HCC still suffer from high tumor recurrence rates, low survival rates, and high resistance rates to cytotoxic drugs [5]. New therapeutic strategies are therefore urgently needed.

Oncolytic virus (OV) therapy represents a promising and innovative immunotherapeutic strategy in oncology. The fundamental principle of OV therapy is to utilize naturally occurring or genetically engineered viruses to selectively replicate in the tumor cells, ultimately leading to the lysis of tumor cells without adversely affecting the surrounding normal cells [6]. Then, the tumor cells release the infectious viral progeny, enabling oncolysis amplification towards neighboring tumor cells [7]. This review is to summarize the mechanism of OVs and the new research advances in their application in HCC, providing us with ideas for the subsequent further exploration of the treatment of HCC. (Our product information and clinical trial information of oncolytic viruses are obtained from the Drug Review Center of the State Food and Drug Administration of China, as well as the U.S. Clinical Trial Registry, American Society of Clinical Oncology and others.)

### The history of oncolytic virus therapy

Oncolytic virus therapy traces its origins to the late 19th century. In 1896, Dock documented a remarkable case of a 42-year-old female with leukemia who experienced remission and tumor regression following an influenza infection [8]. Similarly, another case was reported of a 4-year-old boy whose lymphatic leukemia regressed after a varicella (chickenpox) infection, although it lasted only a month [9]. In the early 20th century, Dr. De Pace in Italy described a case of a woman with cervical cancer who achieved an eight-year remission after being bitten by a dog and subsequently receiving a rabies vaccination. Furthermore, clinical studies have since reported instances of remission in hematological malignancies, including leukemia [10], Hodgkin’s disease [11], and Burkitt’s lymphoma [12], following measles virus infections, often in patients with compromised immune systems. Thus, some investigators have suggested that cancer remission accompanying natural viral infections such as measles and chickenpox can be summarized as follows: occasionally, some viruses can fight tumors by destroying cancer cells; this tumor regression generally occurs in patients with compromised immune systems; and virus-induced remission is short-lived, lasting only for a few months [13]. Motivated by these findings, in 1949, clinical studies were conducted on Hodgkin’s patients infected with hepatitis, resulting in observed antitumor effects in 7 out of 13 participants [14]. Despite these early successes, the use of wild-type viruses in these studies was limited by a lack of understanding of virology and the absence of genetic modification techniques, which raised concerns regarding safety. It was not until the 1990s, with the development of genetic engineering and recombinant DNA technology, that interest in OV therapy was rekindled. Wild-type viruses have been genetically engineered to be more tumor-selective and less pathogenic [15]. The first OV to enter phase I clinical trials was an adenovirus, and the first genetically engineered OV, known as Onyx-015, demonstrated a good safety and tolerability profile, with influenza-like symptoms being the most common adverse effect [16]. Subsequently, in 2005, the Chinese State Food and Drug Administration granted approval for the first recombinant OV, H101 (commercial name: Oncorine), to be used in conjunction with chemotherapy. The approval of H101 marked a significant turning point, sparking exponential growth in clinical research into OV therapy. Today, several OVs have been approved for cancer treatment, underscoring the progress made in this field since the early days of OV research (as shown in Table 1), and more OVs have entered the clinical trial phase.

**Table 1:** Approved Oncolytic Viruses

Oncolytic virus	Approved indication	Approved region
Talimogene laherparepvec (T-VEC) (Imlygic)	Treatment of unresectable metastatic melanoma	USA, Europe
H101(Recombinant Human Type 5 Adenovirus) (Oncorine)	Treatment of late-stage refractory nasopharyngeal cancer	China
Rigvir	Treatment of melanoma	Latvia
DELYTACT	Treatment of glioblastoma and other brain cancers	Japan

### The mechanism of oncolytic virus therapy

OVs are categorized into naturally occurring and genetically engineered and modified transgenic viruses. On the one hand, OVs are able to lyse cancer cells; on the other hand, they can influence the body’s immune response. Most of the viruses selected for research are genetically modified to improve the ability of viruses to selectively replicate and target cancer cells. The principal mechanisms underlying OV therapy include:

**1) Direct tumor lysis.** OV is capable of selectively replicating within tumor cells. After the virus enters the tumor cell, the early gene in the E1 region initiates transcription and translation, and the E1A protein binds to the Rb protein, phosphorylating the Rb protein and releasing the transcription factor E2F, activating the cell cycle to bring the tumor cell into the S-phase, increasing the replication of the virus [17], eventually causing lysis of these cells. This process releases progeny viruses that can then infect neighboring tumor cells, perpetuating a cycle of infection and destruction [18]. OVs can attenuate the antiviral response either through interactions with binding to specific cellular receptors, exploiting inherent defects in tumor cells, or genetically modifying viral vectors to disable genes, enhancing the precision of tumor cell destruction. When the virus enters normal cells, Pathogen-Associated Molecular Patterns (PAMPs) activate the intracellular Toll-like receptor (TLR), which then activates TNF-related factor 3 (TRAF3), together with Retinoic Acid-inducible Gene 1 (RIG-1) activated by viral nucleic acids co-activates downstream factors, further activating the Janus Kinase-Signal Transducer and Activator of Transcription (JAK-STAT) pathway involved in antiviral resistance in infected cells. This pathway promotes an Interferon (IFN)-mediated antiviral response, which together triggers an antiviral response in normal cells, leading to virus clearance [19]. Currently, there are two principal strategies for enhancing the tumor-targeting capabilities of OVs. The first involves augmenting the affinity of the virus for receptors that are overexpressed on tumor cells. The second strategy leverages the aberrant expression of certain pathways or proteins within tumor cells to improve the specificity of viral targeting [20].

**Figure 1:**
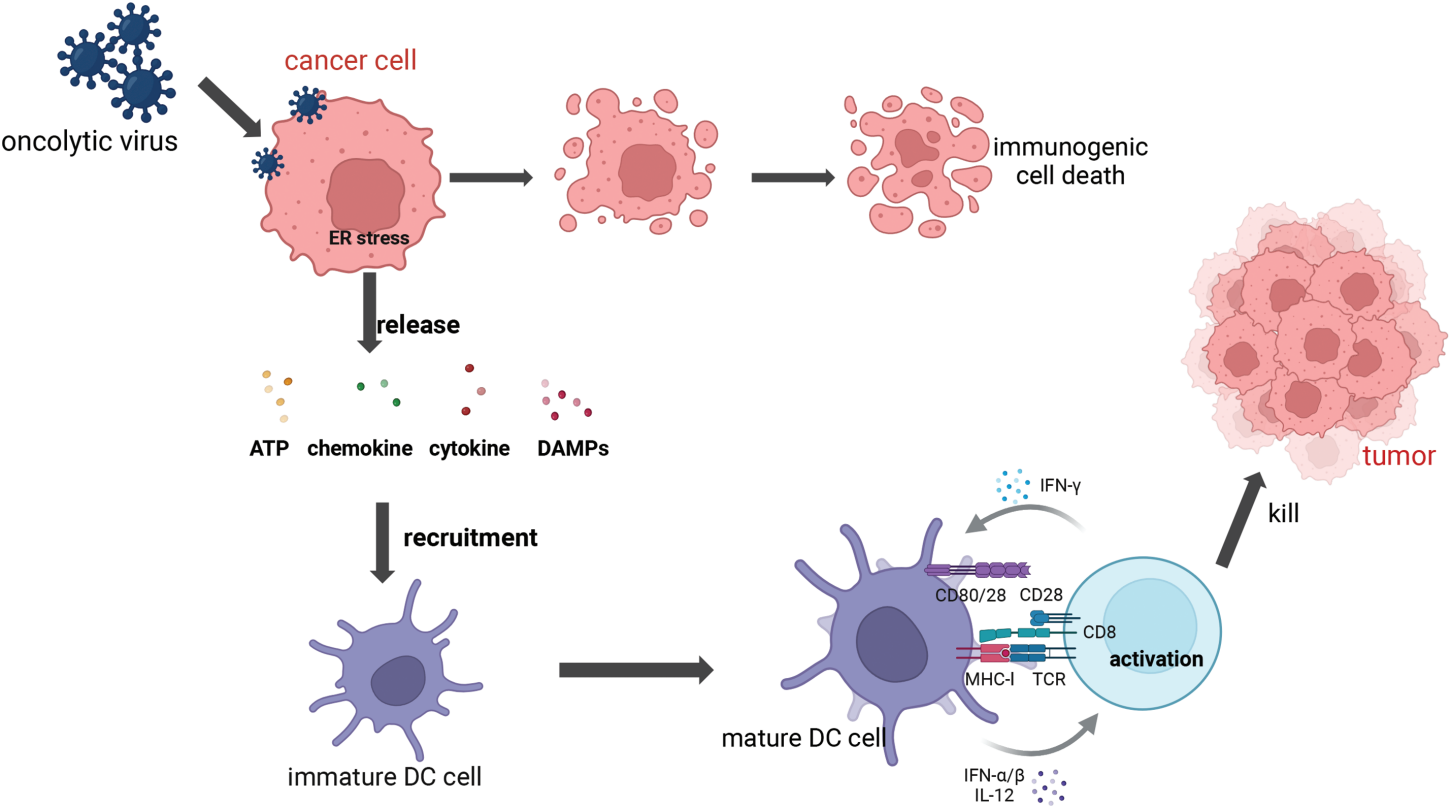
Mechanism of Dendritic Cells (DCs) activation and T cells priming. Following infection with the oncolytic virus, cancer cells initiate an endoplasmic reticulum (ER) stress, which leads to the expression of various damage-associated molecular patterns (DAMPs) such as calreticulin (ecto-CRT) and heat shock proteins (HSP70/90), as well as ATP, cytokine, and chemokine. These substances help to recruit and mature the DC cells, upon recognizing the DAMPs and receiving signals such as IFN-α/β, the DC undergoes maturation, upregulating co-stimulatory molecules like CD80 and CD86, which interact with CD8+ T cells. The T cell receptor (TCR) on the CD8+ T cell recognizes the peptide-Major Histocompatibility Complex (MHC) complex presented by the DC for cell activation to kill cancer cells. Once activated, the CD8+ T cell produces IFN-γ, a cytokine that further enhances the antitumor immune response and can also provide feedback to stimulate DCs (Biorender.com).

**Figure 2:**
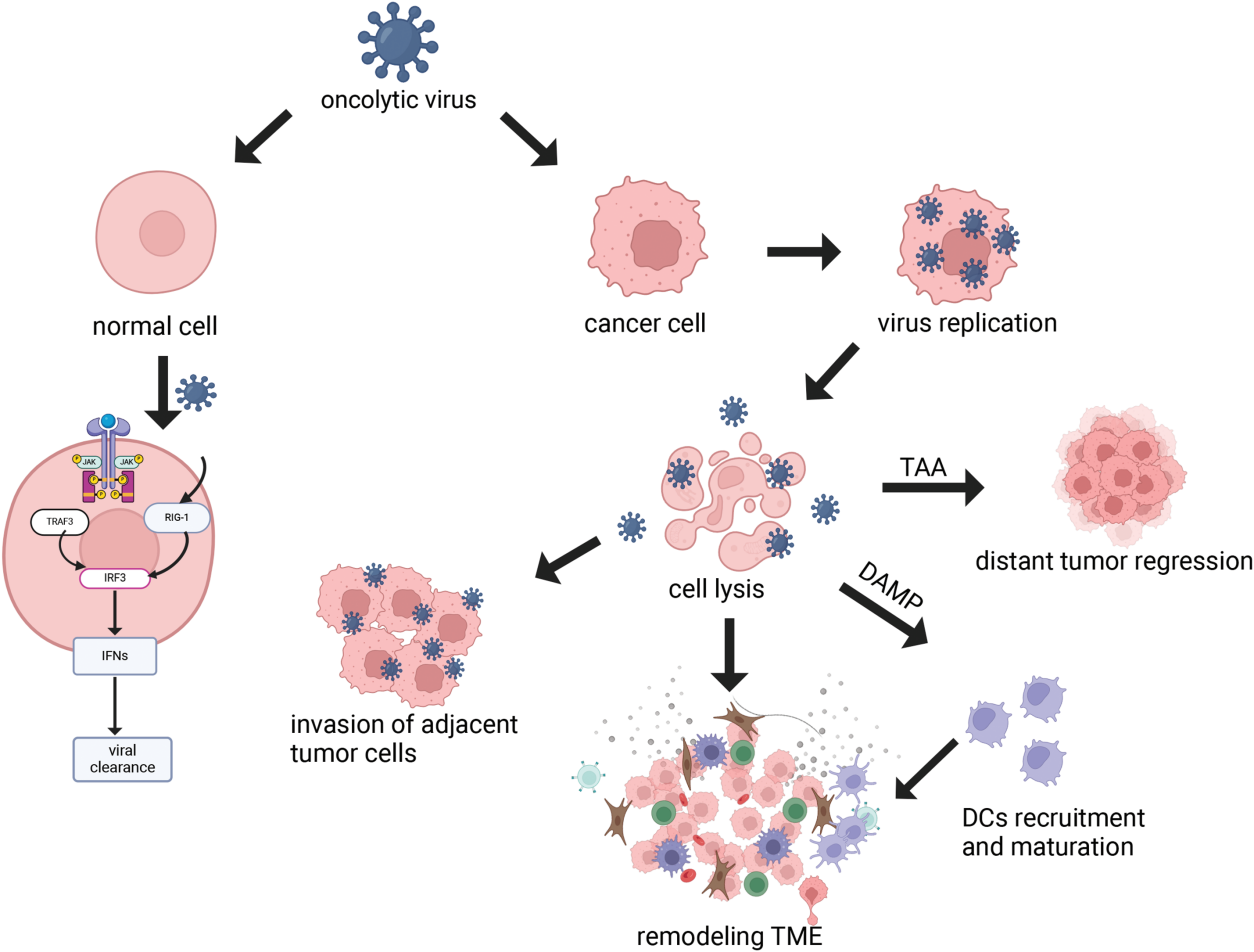
Mechanism of oncolytic virus. OVs can selectively replicate in tumor cells, leading to cell lysis. In normal cells, viral PAMPs activate the Toll-like receptor and RIG-1 receptor pathways to trigger the antiviral mechanism, and this signaling cascade, including JAK, STAT, and interferon, effectively removes the virus and restricts viral dissemination. Viral infection of neighboring tumor cells remodels the tumor microenvironment (TME). Cytolysis releases DAMP, which leads to the recruitment and activation of immune cells such as DCs, thereby aiding in the remodeling of the TME. Tumor-Associated Antigen (TAA) can also be released to kill distant tumors by activating adaptive immune responses (Biorender.com).

**2) Immune activation.** The exposure of tumor cells to OVs results in the generation of Endoplasmic Reticulum (ER) stress and immunogenic cell death (ICD), which are pivotal in the release of damage-associated molecular patterns (DAMPs). These DAMPs encompass the translocation of surface-exposed calreticulin (ecto-CRT) and heat shock proteins (HSP) 70/90 to the cell membrane surface, along with the extracellular release of ATP (Adenosine Triphosphate), chemokines, cytokines, and so on. Furthermore, the recruitment and maturation of immature dendritic cells (DCs) are induced, while the calreticulin ecto-CRT provides phagocytic signals to promote DC phagocytosis of tumor antigens [21]. Additionally, high-mobility group box 1 (HMGB1) and HSP70/90 contribute to phagocytosis by engaging TLRs, which facilitates antigen processing and subsequent presentation to T cells. This cascade of events culminates in the activation of T cells, notably CD8+ cytotoxic T lymphocytes, which then target and destroy tumor cells, thus mediating a tumor-specific immune response [22] (Figs. 1 and 2).

**Figure 3:**
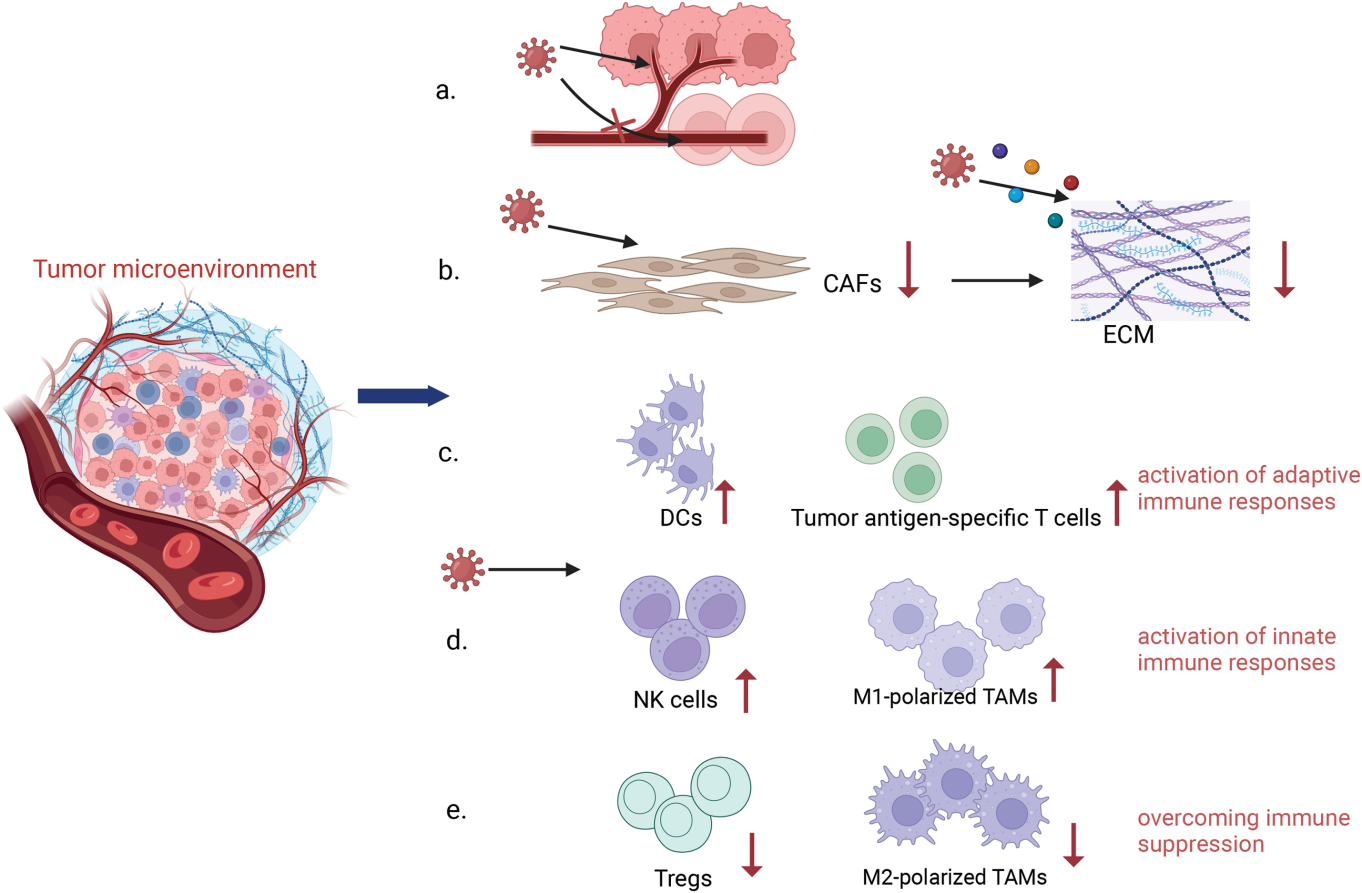
Remodeling Tumor Microenvironment. Oncolytic viruses have the ability to remodel the tumor immune microenvironment. OVs can specifically attack the supply vessels of tumor cells, making tumor cells have no nutritional supply to kill tumor cells, while it has no response to the supply vessels of normal cells (a). OVs can reduce the number of CAFs by infecting them and inducing apoptosis, while OVs are able to target specific components in the tumor stroma, such as collagen, to further reduce the density and stiffness of the ECM and improve the diffusion of viruses within tumors by expressing matrix metalloproteinase (MMP)-1, hyaluronidase and so on (b). OVs infection enhances the infiltration and activity of immune cells within the TME, including innate and adaptive immune cells (c, d). In addition, OVs reduce the number of immunosuppressive cells, such as Tregs, and promote a shift of the immune cells to an anti-tumor state in order to overcome immunosuppression (e) (Biorender.com).

**3) Regulating the Tumor Microenvironment (TME):** The TME is a complex ecosystem comprised of neoplastic cells and a variety of non-transformed cells, including the extracellular matrix, vasculature, and signaling molecules that can foster tumor cell proliferation and metastasis. OVs infection has the potential to modulate the number and activity of immune cells within the TME. This modulation is achieved by reducing the number of immunosuppressive cells and prompting the conversion of immune cells to an anti-tumor phenotype, thereby enhancing the overall immune response [23]. OVs have been observed to promote the infiltration and accumulation of pro-inflammatory cytokines within the TME, creating a more conducive environment for the activation of DCs. Additionally, OVs can release T-cell recruitment factors and chemokines, which increase T-cell infiltration [24]. In a mouse model of hepatocellular carcinoma, treatment with the oncolytic adenovirus was observed to boost cytotoxic T-cell responses against tumors [25]. Furthermore, OVs infection has been shown to promote the conversion of tumor-associated macrophages to the M1 phenotype, characterized by cytotoxic and pro-inflammatory properties, thus augmenting the efficacy of cancer therapy. Furthermore, OVs can modulate the extracellular matrix (ECM), which is predominantly formed by activated cancer-associated fibroblasts (CAFs). The ECM, composed largely of collagenous fibers, creates an impermeable barrier that hinders the effective penetration of OVs within the tumor [26]. In order to overcome this barrier, the tumor cells are able to produce fibroblast growth factor 2 (FGF2), which increases the susceptibility of CAFs to infection with OVs, promoting better targeting of CAFs and disruption of the ECM. By influencing the interactions between CAFs and tumor cells, OVs contribute to the remodeling of the tumor stroma, thereby augmenting the overall antitumor response [27]. OVs also inhibit angiogenesis in tumors by infecting and lysing vascular endothelial cells (VECs) and reducing vascular endothelial growth factor (VEGF) production by tumor cells [28]. VEGF is known to upregulate PRD1-BF1/Blimp1 expression in tumor vasculature through signaling pathways mediated by Erk1/2 and Stat3, thereby increasing the sensitivity of tumor vasculature to Vaccinia virus (OVVs) infection [29]. Engineered OVVs have demonstrated the ability to selectively target and disrupt established tumor vascular systems, offering a strategic advantage in combating angiogenesis in tumors [30] (Fig. 3).

**4) The activation of innate immunity and autoimmunity.** The tumor microenvironment contains immunosuppressive cells, including regulatory T cells (Tregs), myeloid-derived suppressor cells (MDSCs), and tumor-associated macrophages. These cells can inhibit T cell activity by secreting immunosuppressive molecules, such as IL-10 (interleukin 10) and TGF-β (transforming growth factor beta), which hinder the effective elimination of tumor cells by T cells. Moreover, tumor cells within the TME may downregulate or lack the expression of antigens recognizable by T cells, and they may not provide sufficient signals to activate an autoimmune response. This is referred to as a ‘cold’ environment in immunological terms. OVs therapy can alter the TME, and infected cells can release DAMPs such as HMGB1 and HSP, as well as interferon (IFN). These DAMPs stimulate the innate immune response, leading to the recruitment and activation of a plethora of immune cells. This immune activation can transform a ‘cold’ tumor into a ‘hot’ tumor, which is more recognizable to the immune system, thereby initiating the destruction of tumor cells. Studies have indicated that HCC is characterized by low expression of interferon receptors [31]. This deficiency in the IFN pathway not only facilitates the virus’s ability to evade immune clearance but also enhances the efficacy of viral-induced lysis of tumor cells [32]. OVs engage with the IFN system through various mechanisms, which include: (1) the PD-L1/PD-1 axis; (2) the JAK/STAT signaling pathway; (3) the Apolipoprotein B Efflux Carrier (APOBEC) cytosine deaminase family; (4) Nuclear Respiratory Factor 2 (Nrf2); (5) the Toll-like receptor 2/nuclear factor kappa-light-chain-enhancer of activated B cells signaling pathway; (6) Interferon Regulatory Factor 3 [33]. The lysis of tumor cells also leads to the release of tumor-associated antigens (TAAs), which can stimulate the adaptive immune response, potentially targeting distant tumors that have not been directly exposed to the virus [23–25,34,35].

## Some New Developments in the Use of the Oncolytic Virus in HCC

### Newcastle disease virus (NDV)

NDV is a member of the avian paramyxovirus type 1, characterized by a negative-sense, single-stranded RNA genome. Certain NDV strains display tumor-selective replication, lysis, and immunostimulation in nonpermissive hosts, making them promising candidates for oncolytic virus therapy [36]. Although it is infectious in birds, it is relatively less virulent and has a relatively high level of biosafety in humans. Among wild-type NDV strains, NDV/HK84 has demonstrated remarkable efficacy against HCC in both *in vitro* and *in vivo* studies. Findings indicated that NDV/HK84 markedly suppressed the proliferation, migration, and invasiveness of human HCC cell line-SK-HEP-1 cells, outperforming cisplatin (DDP)-positive controls. In a nude mouse xenograft tumor model, intratumoral injections of NDV/HK84 at a dosage of 1 × 10^^^7 EID50 (50% Egg Infective Dose) per 100 μL were administered. After 15 days, complete tumor regression was observed in 60% of the mice, with H&E staining revealing a near-absence of viable tumor cells in the affected tissues [37]. These results underscore the potent oncolytic activity of the NDV/HK84 strain against HCC cells and its favorable safety profile, offering a novel therapeutic avenue for the development of HCC lysis therapies. Future studies should focus on understanding the molecular mechanisms and combining NDV with other therapies to enhance their effects.

## Adenovirus

Adenovirus is a naked virus comprising approximately 35 Kb of linear double-stranded DNA encoding over 40 proteins. Adenovirus is the most common type of virus used in oncolytic therapy due to its capacity for flexible genetic modification, enabling it to infect a diverse range of cell types [38]. Oncorine (also known as H101), the first recombinant oncolytic adenovirus approved by China’s State Food and Drug Administration (CFDA) in 2005, is mainly used for the treatment of nasopharyngeal cancer [39].

### Advances in adenovirus-based therapies

**1) Delivery mediated by mesenchymal stromal cells**

**Lytic effects under hypoxic and normoxic conditions:** Mesenchymal stromal cells (MSCs) possess a remarkable affinity for homing tumors, a trait that has been harnessed to deliver HCC-targeting oncolytic adenoviruses (oAds) directly to the cancerous cells. Studies have confirmed that MSCs loaded with these oAds can efficiently induce the lysis of hepatocellular carcinoma cells under both normoxic and hypoxic conditions. It demonstrated that the MSC-mediated systemic delivery of oAd is a promising approach for antitumor [40] because in the current process of virus delivery, the existing vectors may be taken up and destroyed by the liver and spleen, and may trigger a systemic immune response, causing unnecessary damage.

**Therapeutic effect in combination with immunomodulatory molecules:** Recent studies of many solid tumors have led to the development of BiTE (bispecific T cell engager)-armed oncolytic virus. BiTE is a protein composed of two single-chain variable fragments, one for tumor-associated antigen and the other for CD3 [41]. In a recent study, researchers have engineered a therapeutic system to target liver cancer by combining mesenchymal stem cells (MSCs) with this oncolytic adenovirus that carries a bispecific antibody to PD-L1, an immunosuppressive molecule that is frequently overexpressed in solid tumors, and this BiTE is specifically designed to bind with high affinity to PD-L1. It had a better anti-tumor effect and less liver injury in the orthotopic transplantation model mice. This strategy also improved T cell infiltration and activation in tumor tissues while reducing liver toxicity [42]. The treatment’s efficacy in humans is uncertain as the human tumor microenvironment is more complex and requires further investigation prior to entering clinical trials.

**2) Adenovirus with synthetic gene circuits**

**Effect on tumor cells:** Researchers established a platform for the construction of synthetic oncolytic adenoviruses equipped with synthetic gene circuits [43]. Utilizing this platform, they developed SynOV, an oncolytic adenovirus targeting Alpha-Fetoprotein (AFP)-positive HCC. Experimental data from mouse models, including subcutaneous and orthotopic HCC, revealed that SynOV exerted significant anticancer effects and extended survival times [44].

**Remodeling effect on the tumor microenvironment:** Single-cell RNA sequencing (scRNA-seq) analysis of immune cell dynamics within tumor tissues indicated that SynOV markedly elevated the infiltration of CD8+ T cells, reduced the presence of immunosuppressive regulatory T cells, and fostered a shift toward antitumor M1 macrophages while diminishing CAFs within the TME. Spatial transcriptomics (ST) further elucidated SynOV’s impact on tumor and TME spatial specificity, showing increased infiltration of CD8+ T cells and M1 macrophages in the tumor core, a decrease in the distribution of immunosuppressive cells, and upregulation of apoptosis-related genes in tumor cells post-SynOV treatment. These findings indicate that SynOV remodels the TME through multiple pathways, promoting a shift toward a normalized immune microenvironment. This study offers novel approaches and insights into the mechanisms by which oncolytic viruses can reshape the TME and highlights the potential of SynOV in advancing immunotherapeutic strategies for HCC [45].

**3) Recombinant adenovirus Ad5-ApoA1 regulates cholesterol metabolism**

Cholesterol accumulation in various organs and tissues is a significant factor in the pathogenesis of numerous diseases [46], with many tumor cells exhibiting elevated cholesterol synthesis and uptake [47]. Additionally, cholesterol accumulation within the TME has been linked to increased expression of immune checkpoint genes such as PD-1 and LAG3 on CD8+ T cells [48], leading to T cell exhaustion and diminished antitumor responses. Apolipoprotein A1 (ApoA1) is known for its role in reverse cholesterol transport and anti-inflammatory activities, and reduced ApoA1 levels have been correlated with the progression and metastasis of hepatocellular carcinoma. The recombinant oncolytic adenovirus Ad5-ApoA1 developed in a recent study, which was found to significantly suppress tumor growth and extend the survival of mice in both immunocompetent and humanized immune mouse models. This study further revealed the mechanism of action of Ad5-ApoA1 from molecular and cellular analysis. On the one hand, it enhanced the activity and antitumor efficacy of CD8+ T cells by reducing cholesterol levels in these cells and downregulating PD-1 and LAG-3 expression. On the other hand, it promoted the local infiltration of CD8+ T cells and natural killer (NK) cells within the tumor and improved the tumor immune microenvironment by decreasing intracellular cholesterol levels [49]. This demonstrated that Ad5-ApoA1 could modulate the TIME and enhance the anti-tumor effect through reprogramming of cholesterol metabolism and might be a candidate for HCC treatment.

Adenovirus-based therapies are highly adaptable, enabling the integration of immune-modulating molecules and tumor-specific targeting systems. Future study needs to optimize delivery methods, explore combination strategies with metabolic reprogramming agents, and solve the translation problem from animal models to clinical applications.

## Herpes Simplex Virus

Herpes simplex virus is a virus composed of a 150-kb double-stranded DNA genome characterized by repetitive sequences. Since the approval of Talimogene laherparepvec (T-VEC) by the U.S. Food and Drug Administration (FDA), an HSV-based OV, oncolytic virotherapy has gained significant attention as a potential cancer treatment in numerous studies [50]. Although T-VEC was initially approved for use in melanoma, its efficacy is also being evaluated in liver cancer.

### Research on improving the oncolytic effect

**1) Combination therapy strategies**

According to a study, it was discovered that the combination of T-VEC and intratumoral radiofrequency hyperthermia (RFH) could enhance the oncolytic effect of T-VEC. After treating HCC in mouse models with the combination therapy mentioned above, it was found that the survival rate of HCC cells decreased in both *in vivo* and *in vitro* experiments. Furthermore, the tumor volume in the combined treatment group was the smallest in the *in vivo* experiment [51]. This also offers a novel concept for the ongoing investigation into the therapeutic potential of Herpes Simplex Virus type 1 (HSV-1) as an oncolytic agent.

**2) Strategies to overcome immunosuppression**

**Combination with IDO1 Inhibitor:** Indoleamine 2,3-dioxygenase 1(IDO1), an important immunosuppressive protein, has been found to be upregulated in tumors. IDO1 can suppress the activation of CD8+ T cells and NK cells within the tumor microenvironment while simultaneously enhancing the recruitment of MDSCs, thereby inhibiting the replication of HSV-1 [52]. Recent research indicates that the combination of Navoximod, a selective IDO1 inhibitor, with HSV-1 significantly enhances the oncolytic efficacy of HSV-1. Researchers utilized an injectable hydrogel as a vector to ensure the replication and distribution of the virus within the tumor tissue. This study showed that the combination of Navoximod and HSV-1 through silk-hydrogels improves the survival time of HCC-bearing mice and inhibits tumor recurrence, and also showed an effective therapeutic efficacy in the orthotopic liver cancer model of rabbit [53].

**Combination with Nanobody:** Researchers have developed a single-domain camel nanoantibody B7H3nb with high affinity for B7H3, a tumor-associated antigen that is overexpressed in various cancers and underexpressed in normal tissues [54,55], and combined it with a single-chain variable fragment (scFv) targeting CD3 to create a bispecific antibody, B7H3nb/CD3, which significantly enhanced cytotoxic activity by redirecting T cells to B7H3-expressing tumor cells. Building on prior research that constructed antibodies with increased selectivity and reduced neurotoxicity to tumors by deleting the ICP6, ICP34.5, and ICP47 genes in HSV-1 (HSV-1dko) [56], the researchers inserted the BsAb (B7H3nb/CD3) gene into the HSV-1 genome using CRISPR/Cas9 technology, resulting in HSV-1dko-B7H3nb/CD3. This engineered virus was designed to limit cytotoxicity to tumor cells, thereby maximizing anticancer effects while minimizing systemic toxicity. The virus demonstrated its ability to significantly increase T-cell infiltration, reduce immunosuppressive cells, and markedly improve antitumor efficacy both *in vitro* and in animal models of glioblastoma and colon cancer in mice [57]. This approach illustrates the effectiveness of combining the HSV-1 oncolytic virus with nanobody-based bispecific antibodies to overcome the challenges posed by the immunosuppressive microenvironment in solid tumors. The high tumor specificity and immunomodulatory capacity of this engineered HSV-1 virus make it applicable in the complex immune microenvironment of HCC.

HSV offers a robust platform for genetic engineering, while its efficacy in the immunosuppressive HCC microenvironment remains limited. The stability of the nanobodies opens up new possibilities for targeted therapies for hepatocellular carcinoma. Future studies should combine HSV with nanobodies or Immune Checkpoint Inhibitors (ICIs) to overcome these challenges.

### Measles virus

The measles virus (MV) is a negative-sense, single-stranded RNA virus with a genome of approximately 16 kilobases in length. Since the development of the live-attenuated measles vaccine from the Edmonston strain, it has been propagated in human kidney cells and chicken embryos. Presently, we possess highly safe, attenuated measles vaccines [39], which have rendered the measles virus an increasingly favorable candidate for oncolytic applications [58]. With our understanding of the chemicals of plants, some researchers have found that natural plant chemicals also have anti-cancer effects that can complement OV therapy. Notably, ursolic acid (UA) has been demonstrated to enhance the oncolytic potency of MV on breast cancer cells [59]. Preclinical studies have also explored the use of UA for the treatment of HCC. It has been observed that UA can suppress the proliferation of HCC cells, induce cell cycle arrest, and promote apoptosis, thereby inhibiting tumor growth [60]. In a recent study, the combination of UA and the measles virus was investigated for its effects on liver cancer cells. The study compared cells treated with UA post-infection to those treated separately with MV and UA. The combined treatment was found to significantly reduce cell survival rates and enhance cell-killing abilities. By assessing the cleavage levels of Poly (ADP-Ribose) Polymerase (PARP), a marker of apoptosis, the study confirmed that the apoptotic rate in cells co-treated with UA and MV was markedly higher, particularly in the early and late stages of apoptosis. This increase in apoptosis led to more cell deaths. Overall, the study demonstrated that UA could enhance the oncolytic activity of MV against liver cancer cells, leading to increased cancer cell mortality through multiple mechanisms [61].

MV’s combination with natural compounds like UA highlights the potential of integrating oncolytic virotherapy with complementary therapeutic agents. It provides a new idea for us to explore other combinations with synergistic effects and mechanisms.

## Reovirus

Reovirus is one of the naturally occurring OV, and its natural strain recognizes altered signaling pathways in cancer without the need for genetic engineering [62]. It had been found that in a tumor model associated with Hepatitis C virus (HCV)-induced HCC, reovirus can inhibit HCV replication by triggering a proinflammatory response mediated through the activation of type I interferons. Additionally, reovirus has been observed to activate innate immune cells, thereby eliciting antitumor effects and effectively combating Hepatitis B virus (HBV)-driven HCC. This research demonstrates the dual utility of reovirus therapy, which is not only antitumor but also capable of targeting oncogenic viral infections [63], a significant consideration given that HCC is predominantly linked to hepatitis viral infections.

**Evaluation of Infection Efficiency:** Researchers investigated the efficacy of reovirus in infecting cancer cells derived from HCC biopsies. They initially determined the multiplicity of infection (MOI) of the virus by employing the plaque assay, a method that quantifies viral infectivity. Subsequently, they assessed the impact of reovirus infection on the cancer cells by examining viral RNA replication, protein synthesis, and cellular viability.

**Verification of Oncolytic Effect:** The study demonstrated that reovirus can replicate extensively in tumor cells derived from HCC biopsies and eventually destroy the cancer cells [64], validating the potential of reovirus as a therapeutic agent in the treatment of HCC. In addition, reovirus has entered a systemic therapeutic trial, and no dose-limiting toxicity was observed in 18 patients with advanced solid tumors given a viral dose of 3 × 10^^^10 TCID50 (Tissue Culture Infective Dose 50%). Even when multiple reovirus injections were given on successive days, grade two adverse events occurred in only 2 patients [65]. This suggests that reovirus is very well tolerated by patients when administered intravenously with the eutherian virus.

Reovirus shows its unique advantages and potential, especially in targeting hepatitis virus-related HCC, making it a unique therapeutic candidate. However, its efficacy in human HCC requires further validation, particularly in combination with systemic immunotherapies.

### Influenza virus

The genome sequence, protein structure, and function of influenza viruses have been well studied, and the ability to induce strong immune responses while improving safety through genetic engineering has attracted widespread attention in the field of tumor immunity. Cytotoxic T-lymphocyte-associated protein 4 (CTLA4) is a crucial immune checkpoint inhibitor present on T cells. The combination of antigen-presenting cell B7 with CTLA4 on T cells inhibits the function of T cells and suppresses autoimmunity. Anti-CTLA4 antibodies, on the other hand, can block this process to activate T cells [66].

**1) Chimeric oncolytic virus carrying CTLA4 antibody:** Previous studies developed a chimeric oncolytic virus incorporating a murine-derived CTLA4 antibody, rFlu-huCTLA4, which has demonstrated tumor growth inhibition in a mouse model of HCC [67]. However, due to the low sequence homology between human and murine CTLA4 antibodies, researchers have recently engineered a chimeric oncolytic virus strain carrying the human CTLA4 antibody, rFlu-huCTLA4. This chimeric virus exhibited significantly higher selective cytotoxicity in HCC cell lines (HepG2 and Huh7) compared to normal hepatocytes, as shown by crystal violet analysis. Furthermore, intratumoral injection of this chimeric virus was found to inhibit tumor growth and extend the survival of mice in animal models. A notable increase in CD4+ and CD8+ T cells was observed in tumor-bearing mice treated with the chimeric virus, indicating that the chimeric virus rFlu-huCTLA4 can selectively target and kill HCC cells both *in vivo* and *in vitro* [68].

**2) Recombinant influenza virus expressing PD-L1 antibody:** A recombinant oncolytic influenza virus, designated rgFlu/PD-L1, was engineered to express an antibody targeting PD-L1. Its expression on tumors binds to the corresponding receptor on T cells, thereby inhibiting T cell activity and diminishing anti-tumor effects. The study revealed that this recombinant virus effectively suppressed PD-L1 expression in HepG2 cells and triggered apoptosis. Furthermore, it was demonstrated that rgFlu/PD-L1 modulates the activity and function of CD8+ T cells by activating the cGas-STING pathway, thereby augmenting the cytotoxicity against HCC cells [69].

**3) Recombinant influenza virus carrying the GV1001 sequence:** GV1001 is a peptide derived from human telomerase reverse transcriptase, which is highly expressed in tumor cells since most malignant tumors require telomerase activity to maintain cellular immortality [70]. This peptide vaccine has been shown to reduce the expression levels of intracellular heat-shock proteins, thereby enhancing its antitumor effects [71]. A clinical trial has investigated the combination of GV1001 with chemotherapeutic agents in colorectal cancer patients who have failed first-line chemotherapy, demonstrating a dose-dependent tolerability and potential efficacy [72]. Building on these findings, researchers have constructed a chimeric recombinant influenza virus carrying the GV1001 sequence, named HSV-1dko-B7H3nb/CD3, to leverage the therapeutic benefits of both OV and the antitumor effects of GV1001. In a mouse liver cancer model, intratumoral injection of this virus at a dose of 1 × 10^^^4 TCID50 and 1 × 10^^^6 TCID50 demonstrated high selectivity for tumor cells and a significant safety profile. Flow cytometry analysis of splenic lymphocytes revealed an increased number of CD4+ and CD8+ T cells in the treatment group compared to the control, indicating that the recombinant virus could induce tumor cell death through the activation of cellular immunity [73]. The persistence of the recombinant influenza viruses and GV1001 and the mechanism of killing in tumor therapy still need to be further investigated.

### Vaccinia virus

Oncolytic Vaccinia Virus (OVV) is a double-stranded DNA virus with a genome of approximately 190 kb, capable of accommodating and expressing large exogenous genes. OVV is known for its rapid and lytic replication cycle, which upon host invasion, can swiftly initiate replication and proliferate extensively, triggering robust immune and inflammatory responses [74]. In recent years, OVV like JX-594 (Pexa Vec), an oncolytic virus carrying granulocyte-macrophage colony-stimulating factor (GM-CSF), has shown promising results in clinical trials and is among the most extensively tested OVV. OVVs have demonstrated antitumor effects and a favorable safety profile in various solid tumors, used either as monotherapy or in combination with other treatments [75]. Another example is JX929, an OVV modified to selectively replicate in tumor cells by altering the VGK (Vaccinia virus G protein) and TK (Thymidine kinase) genes, which was safely administered intravenously in a phase I clinical trial for multiple solid tumors without observing dose-limiting toxicity [76,77].

Lectins, a class of non-enzymatic, non-antibody sugar-binding proteins, particularly those of marine origin, have shown the ability to induce apoptosis, autophagy and inhibit angiogenesis in cancer cells [78]. Researchers have investigated the OVV, which carries Aphrocallistes vastus lectin (AVL) in liver cancer. *In vitro* studies with Huh7, Hep-3B, and SK-Hep-1 cells infected with oncoVV (oncolytic vaccinia virus) and oncoVV-AVL showed decreased viability and increased apoptosis in infected HCC cells over time, with upregulated expression of caspase-8 and cleaved-caspase-3, indicating anti-proliferative and pro-apoptotic effects. In a mouse subcutaneous tumor model, oncoVV-AVL significantly inhibited tumor growth, promoting nuclear fragmentation and coagulative necrosis in tumor cells [79]. OncoVV-ALV showed significant replicative and anti-tumor effects *in vitro* and *in vivo*, but the specific mechanisms were not elucidated. Additionally, in the team’s latest study, metabolomics analysis, RT-qPCR, and other experiments revealed that oncoVV-AVL-infected cells exhibited increased α-Ketoglutaric acid (α-KG), Nicotinamide adenine dinucleotide (NADH), and ATP levels, and decreased NADP+/NADPH ratios, suggesting that oncoVV-AVL may inhibit tumor growth by enhancing oxidative phosphorylation and modulating the NADP+/NADPH ratio, potentially increasing ROS(Reactive Oxygen Species) production and promoting lipid synthesis to enhance viral replication through the ROS/NRF2/FASN signaling pathway [80].

In conclusion, oncoVV-AVL may promote ROS production by reprogramming HCC cell metabolism, with the increased ROS further promoting viral replication and inducing apoptosis. Given the metabolic effects of oncoVV-AVL, future studies could consider combining it with metabolically targeting drugs to enhance the therapeutic efficacy against HCC.

Related OVs and their major mechanisms are shown in Table 2. Relevant OVs and their clinical trials are shown in Table 3.

**Table 2:** Oncolytic viruses and their related major mechanisms

Type	Mechanism
NDV	Dependent on activation of type I interferon signaling [37]
Adenovirus	Induces cell apoptosis and inhibits tumor metastasis [40]
HSV	Promotes the activation of T cells and enhances the oncolytic ability of the virus [51]
MV	Promotes ROS accumulation, causes oxidative stress, and leads to apoptosis [61]
Reovirus	Activates type I interferon [63]
Influenza virus (Chimeric virus)	Enhances selective cytotoxicity and the number of T cells [68]
Vaccinia virus	Act through viral replication and induction of immunity [74]

**Table 3:** Clinical trials of OVs for HCC

Oncolytic virus	Trial number	Status	Combination	Trail phase	Time
**Adenovirus**					
H101	NCT05113290	Unknown	Sorafenib	IV	2021
H101	NCT06253598	Not yet recruiting	Lenvatinib	II	2024
			Tislelizumab		
H101	NCT06685354	Not yet recruiting	alone	II	2024
H101	NCT05872841	Not yet recruiting	TACE	II	2023
SynOV1.1	NCT04612504	Recruiting	Alone	I	2022
H101	NCT05675462	Recruiting	Tislelizumab	I	2023
			Lenvatinib		
H101	NCT03563170	Withdrawn	ETBX-011	I, II	2018
			GI-4000		
H101	NCT03780049	Unknown	HAIC of FOLFOX	III	2018
rAd-p53	NCT03544723	Unknown	Alone	II	2018
H101	NCT03790059	Unknown	RFA (Radiofrequency Ablation)	Not applicable	2016
rAd-p53	NCT02561546	Unknown	Trans-catheter embolization	II	2015
rAd-p53	NCT02509169	Unknown	TAE	II	2014
rAd-p53	NCT02418988	Unknown	TACE	II	2014
ADV-TK (Aglatimagene besadenovec)	NCT03313596	Unknown	Alone	III	2013
H101	NCT01869088	Unknown	TACE	III	2013
ADV-TK	NCT02202564	Completed	Ganciclovir	II	2006
Ad-HSVtk	NCT00844623	Completed	Alone	I	2002
ADV-TK	NCT00300521	Completed	Alone	II	2000
Ad5CMV-p53 gene	NCT00003147	Terminated	Alone	I	1998
**Herpes simplex virus**					
RP2	NCT05733598	Recruiting	Bevacizumab	II	2024
			Atezolizumab		
VG161 (Human IL12/15-PDL1B)	NCT06124001	Not yet recruiting	Camrelizumab	I	2023
VG161	NCT04806464	Not yet recruiting	Alone	I	2022
KB707	NCT06228326	Recruiting	Alone	I	2024
T-VEC	NCT02509507	Completed	Pembrolizumab	I, II	2016
**Vaccinia virus**					
GC001	NCT06508307	Recruiting	Alone	I	2023
PF-07263689	NCT05061537	Terminated	Sasanlimab	I	2021
AFM13	NCT04124895	Recruiting	Alone	I, II	2020
Pexa Vec	NCT02562755	Completed	Sorafenib	III	2015
Pexa Vec (TK-deletion plus GM-CSF)	NCT00554372	Completed	Alone	II	2008
Pexa Vec	NCT01387555	Completed	Alone	II	2008
Pexa Vec (TK-deletion plus GM-CSF)	NCT00629759	Completed	Alone	I	2006
**M1 virus**					
M1-c6v1	NCT04665362	Unknown	Apatinib	I	2020

## The Combination Medication for Oncolytic Virus

### Oncolytic virus in combination with immune checkpoint inhibitors

Immune checkpoint inhibitors (ICIs) enhance the body’s anti-tumor immune response by alleviating the immunosuppressive state of the tumor microenvironment. Nivolumab, a monoclonal antibody targeting PD-L1, received approval in 2017 for the immunotherapy of advanced HCC. A recent clinical study has shown that the efficacy of nivolumab is not compromised by prior sorafenib treatment, with an objective response rate of 23% and a 9-month overall survival rate of 82% in untreated patients, which supports the consideration of nivolumab as a first-line treatment option for patients with advanced HCC [81]. Additionally, the U.S. Food and Drug Administration has approved the combination therapy of Nivolumab with Ipilimumab as a second-line treatment for HCC [82]. A team has already conducted a comprehensive analysis of individual T cells in six hepatitis B virus-positive HCC patients using single-cell sequencing technology, which is expected to explore new immune checkpoint targets for HCC [83].

However, treatment with single-agent ICIs faces two major challenges. Firstly, about 5%–20% of patients receiving immunotherapy experience serious adverse effects; about 10%–20% of patients experience serious immune-related adverse events (irAEs) [69,84]. Secondly, the objective remission rate is low, and the disease control rate is unsatisfactory. Several single-arm studies of PD-1/PD-L1 inhibitors have shown that although the objective remission rate is better in HCC, the disease control rate for single-agent therapy is not as high in advanced HCC [85]. This has prompted investigators to explore new strategies for combining ICIs with OVs.

The synergistic mechanisms between OVs and ICIs are mainly reflected in four aspects. Firstly, researchers have found that the presence status and infiltration of immune cells within a tumor affects the tumor’s response to immune checkpoint blockade therapy [86], and OV therapy has the potential to reshape the TME by enhancing the infiltration of immune cells into the tumor, which may help to restore the sensitivity to ICIs, thereby improving the therapeutic efficacy of ICIs and enhancing anticancer activity [87]. Secondly, OVs can activate and expand antitumor T cells, which, when further potentiated by ICIs, can elicit more robust antitumor effects than either treatment alone. Thirdly, after infection of cancer cells, OVs can activate PD-L1 expression in tumor cells by releasing virus-associated pattern molecules (e.g., PAMPs) and pro-inflammatory factors (e.g., IFN-γ), thereby rendering tumors that are otherwise unresponsive to PD-L1 blockade potential responders [88]. Finally, OVs have been demonstrated to overcome tumor resistance to immune checkpoint inhibitors. This integrated strategy aims to harness the complementary mechanisms of action of both modalities to enhance the overall efficacy of cancer immunotherapy [89,90].

However, not all OVs in combination with ICIs produce superior anticancer effects. Previous studies have shown that the addition of interferon-β-expressing VSV (Vesicular Stomatitis Virus)-IFNβ to anti-PD-L1 therapy reduced the therapeutic effect. VSV-IFNβ significantly amplified the antiviral response within the CD8+ T-cell compartment, which corresponded to a relative reduction in the population of antitumor T-cells targeted by anti-PD-L1 therapy [91,92]. This suggests that the high immunogenicity of viral therapies might preferentially expand the antiviral T-cell population, potentially inhibiting the antitumor immune response. Consequently, researchers have attempted to incorporate tumor-associated antigens into VSV to create chimeric viruses capable of inducing synergistic antiviral and antitumor immune responses. Utilizing the Sleeping Beauty transposon system, they generated a multifocal hepatocellular carcinoma model in mice. In this model, it was observed that when VSV encoded tumor antigens, virotherapy enhanced antitumor CD8+ T-cell responses, and the combination of ICI and chimeric viruses achieved 100% long-term survival in some experimental groups [93]. This demonstrates that chimeric viruses expressing tumor antigens can redirect antiviral immunity towards antitumor immunity, leading to more effective tumor clearance.

Future optimization in immunotherapy may focus on the following areas. On one hand, identifying new immune checkpoint targets such as CTLA4 or analyzing the T cell profiles. On the other hand, engineering OVs to induce neoantigen expression in tumors.

### Oncolytic virus combined with small molecule inhibitors

Sorafenib received approval for systemic therapy of advanced HCC in the European Union and the United States in 2007 [94]. In recent years, some novel molecularly targeted agents such as apatinib and regorafenib have also been approved as second-line therapeutic agents [95]. However, similar to chemotherapy, these drugs are often associated with a high incidence of resistance and significant side effects, which limit their clinical efficacy, and combination with OVs is a novel approach to overcome these limitations through a multi-targeting mechanism.

**Angiogenesis Targeting:** Sorafenib, a small molecule inhibitor, exerts its action by inhibiting vascular endothelial growth factor (VEGF) signaling in HCC cells. When used in conjunction with OVs, it can enhance the infectivity of the tumor vasculature. Prior research has shown that the oncolytic virus JX-594 increases tumor susceptibility to subsequent treatment with VEGF inhibitors [96]. However, a recent phase III clinical trial indicated that the sequential administration of JX-594 and sorafenib did not confer additional clinical benefits in advanced HCC and, in fact, performed poorer than sorafenib monotherapy [97]. This latest study, which was based on a larger sample size and employed a more rigorous design that controlled for confounding variables, may provide a more accurate reflection of the true efficacy of such combinations. These findings suggest that while combination therapy has shown promise in preclinical settings, the translation of this combinatorial approach to clinical practice may require optimized timing and dosing [98].

**Metabolic Reprogramming:** It is a characteristic of tumors, especially glycolysis. As shown by the Warburg effect, even in the presence of sufficient oxygen, cancer cells still tend to break down glucose into lactate for cell proliferation [99]. Therefore, targeting glycolytic enzymes overexpressed in tumors may be a subsequent breakthrough point in our study of combination therapies. Acylphosphatase 1 (ACYP1), a small cytoplasmic protein, has been shown to boost the metabolic capacity of tumor cells by promoting glycolysis, thereby enhancing tumor proliferation, invasion, and migration. In a study investigating the potential of targeting ACYP1 in combination with levatinib, a first-line therapy for advanced HCC, it was found that this dual targeting significantly increased HCC sensitivity to levatinib and slowed tumor progression [100]. This suggests the potential for developing metabolism-targeted oncolytic viruses that preferentially infect cells with high ACYP1 expression, further improving the specificity of the virus in HCC and enhancing the tumor killing ability.

**Drug Resistance Modulation:** Recent studies have indeed explored various aspects of resistance, including alterations in molecular markers, activation of signaling pathways, and epigenetic changes. A notable study has specifically examined the role of microRNAs (miRNAs) in sorafenib resistance at the genomic level. The results showed that miR-3689a-3p overexpressing tumors were more sensitive to sorafenib treatment, while miR-3689a-3p knockdown tumors grew rapidly even in the presence of sorafenib. This suggests that we can subsequently target miR-3689a-3p to further improve our current therapeutic strategy [101]. Another study has delved into the mechanisms of sorafenib resistance, particularly in relation to iron death, a form of regulated cell death associated with iron metabolism dysregulation. Sorafenib is thought to induce iron death through the inhibitory system Xc-, which mediates cystine inputs, ultimately leading to glutathione depletion and the accumulation of iron-dependent lipid reactive oxygen species (ROS) in cancer cells, thereby generating an anticancer effect [102]. Whereas, researchers initially assessed the expression levels of Dual-Specificity Phosphatase 4 (DUSP4) in HCC cells by analyzing public datasets from HepG2 and Huh7 cell lines and monitoring expression changes post-sorafenib treatment. It was then illustrated by qRT-PCR, WB, and a nude mouse model that DUSP4 inhibition enhanced tumor sensitivity to sorafenib, as demonstrated by tumor growth inhibition [103]. This research suggests that DUSP4 acts as a negative regulator in sorafenib-induced iron death in cancer cells. These findings provide the rationale for engineering OVs to target resistance mechanisms.

This therapy offers hope against drug resistance and enhances treatment, but challenges remain in applying it clinically. Further research is needed to understand how drug combinations, viral factors, dosage, and timing affect treatment. Future work should focus on developing new small molecule inhibitors with new targets, combining them with OVs, and bridging preclinical research with clinical trials to speed up clinical application.

### Oncolytic virus combined with chemotherapy

Chemotherapy is one of the commonly used treatments for cancer, and the most commonly used are cytotoxic drugs that work primarily by acting on proliferating cells and inhibiting their DNA replication and cell cycle. Conventional chemotherapy is limited by its non-specific cytotoxicity, poor tumor penetration, and the potential for further liver injury. However, several studies in the past few years have shown that the combination of OVs with chemotherapeutic agents can produce a synergistic effect, increase the sensitivity of cancer cells, modify the tumor microenvironment, and enhance the therapeutic effect [104].

A breakthrough in this combination came with a novel recombinant poxvirus, OVV-Hyal1, which can re-engineer the TME by degrading hyaluronic acid (HA), thus promoting the dispersion of the virus and the permeation of chemotherapeutic drugs throughout the tumor. In a mouse model of subcutaneous pancreatic cancer, OVV-Hyal1 at a dose of 2 × 10^^^7 pfu (plaque forming units) was found to enhance the distribution and concentration of doxorubicin and gemcitabine within the tumor, significantly amplifying their antitumor effects. Notably, among the eight mice treated with OVV-Hyal1 in combination with gemcitabine, two achieved a complete response, and three achieved a partial response, with a marked increase in cancer cell apoptosis compared to gemcitabine alone [105]. These findings suggest that OVV-Hyal1 warrants further investigation in clinical trials for solid tumors, including HCC.

Futher molecular insights into the synergy between OVs and chemotherapy have been revealed through the study of E4orf6-deleted oncolytic adenovirus combined with cisplatin. HuR, an RNA-binding protein, interacts with the AU-rich element (ARE) of specific mRNAs, playing a role in mRNA stabilization and nucleation. It has been shown that cisplatin upregulates HuR expression in the cytoplasm. The combined therapy was found to augment viral replication by upregulating CDDP(Cis-Diamminedichloroplatinum)-induced cytoplasmic HuR, leading to the stabilization of ARE mRNA, while also triggering the intrinsic apoptotic pathway and amplifying cancer cell death [106].

In summary, the combination not only addresses the limitations of conventional chemotherapy but also offers a novel therapeutic strategy for the exploration of HCC treatment options.

### Oncolytic virus combined with locoregional therapies

The integration of OVs with locoregional therapies, particularly radiofrequency ablation (RFA) and transarterial chemoembolization (TACE), represents an innovative approach to overcome the limitations of conventional treatments for HCC. The clinical efficacy of radiotherapy is attributed to direct tumor cell killing effects induced by DNA damage [107] as well as local and even distant tumor control mediated by enhanced tumor-specific immunity [108]. RFA is considered to be the standard local treatment for liver cancer. However, the biggest problem of RFA is that although it can eliminate the primary lesions in the liver, it can easily lead to the generation of metastatic lesions in the liver.

The combination of OVs with radiotherapy (RT) has demonstrated particularly compelling results through multiple complementary mechanisms, which bolster tumor-specific immune responses and counteract the immunosuppressive effects of RT in HCC [109]. Firstly, OVs can suppress the repair of DNA damage following radiotherapy and enhance radiosensitivity by inhibiting critical repair proteins [110]. At the same time, radiotherapy induces apoptosis and releases a large number of TAAs and DAMPs, thus promoting the replication and dissemination of OVs. RFH also enhances the delivery of the oncolytic virus by intratumoral injection, thereby reducing the toxicity of administration through the vein [51]. In a landmark study utilizing the herpes simplex virus G47Δ (2 × 10^^^6 pfu) in combination with RFA, researchers observed significant reductions in contralateral tumor growth and enhanced systemic immune responses by a syngeneic Neuro2a mouse model. Flow cytometry analysis revealed an increase in CD8+ T cells within the tumor, and the neutralization of the combination therapy’s enhanced antitumor effect by anti-CD8 monoclonal antibody confirmed the pivotal role of CD8+ T cells. This preclinical study of oncolytic virotherapy in conjunction with RFA may reveal that the thermal stress induced by RFA enhanced viral replication and spread within the TME, creating a synergistic effect to suppress the emergence of untreated or new intrahepatic foci by inducing specific antitumor immunity [111].

Similarly, the combination of OVs with TACE has emerged as a promising strategy to address the limitations of conventional TACE therapy. For HCC, TACE induces apoptosis and necrosis in most tumor cells due to local hypoxia. However, hypoxia also upregulates hypoxia-inducible factor-1α (HIF-1α), which can foster tumor recurrence or metastasis. To target residual tumor cells expressing HIF-1α, researchers have developed a synthetic hypoxia-activated replicating adenovirus (HYAD). These modified viruses preferentially replicate in hypoxic conditions, targeting residual tumor cells that often survive traditional TACE treatment. In a VX2 rabbit model of HCC, HYAD at a dose of 1 × 10^^^9 pfu with polyvinyl alcohol embolization has demonstrated improved tumor control rates and reduced incidence of metastatic spread compared to standard TACE procedures [112].

In a recent study, researchers identified MELK (maternal embryonic leucine zipper kinase) as a potential driver of HCC tumorigenesis and a biomarker for poor prognosis. They developed a nude mouse tumor xenograft model using the MELK-knockdown HCC cell line and observed significantly reduced tumor sizes compared to the control group. Notably, the combination of MELK knockdown with radiotherapy demonstrated an enhanced antitumor effect, suggesting a synergistic interaction between MELK inhibition and radiotherapy [109]. This finding offers a promising new target for HCC treatment, paving the way for the development of recombinant oncolytic viruses through genetic engineering.

The success of these combination approaches may rely on the timing and sequence of treatments. This may be because the expression of HSPs following RFA can facilitate viral entry and replication, while the temporary disruption of tumor vasculature after TACE builds an optimal condition for viral delivery and spread.

### Oncolytic virotherapy combined with CAR-T cell therapy

**Figure 4:**
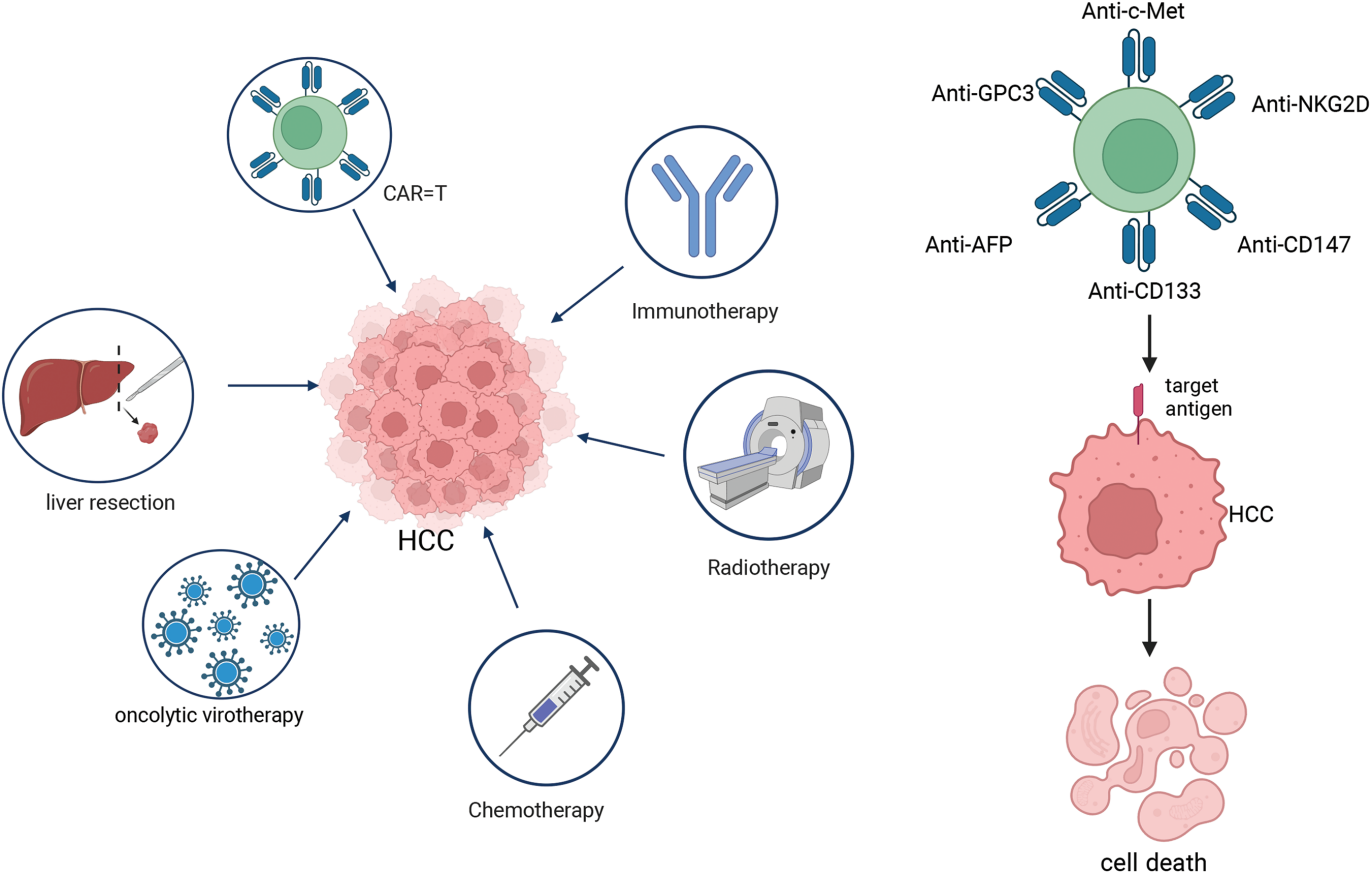
Treatment method of hepatocellular carcinoma (HCC). The treatment methods for HCC include operative treatment (such as liver resection), oncolytic virotherapy, chemotherapy, radiotherapy, immunotherapy, and CAR-T cell therapy. CAR-T cell therapy exhibits both diversity and specificity, with each type of CAR-T cell targeting a distinct antigen on HCC. Notably, GPC3-targeted CAR-T cells, CD133-targeted CAR-T cells, c-Met-targeted CAR-T cells, NKG2D-targeted CAR-T cells, AFP-targeted CAR-T cells, and CD147-targeted CAR-T cells have demonstrated significant efficacy in the treatment of HCC.

CAR-T therapies are immunotherapies that are being actively developed for the treatment of malignant tumors. Due to the major breakthroughs in the treatment of lymphomas, CAR-T therapy has been explored for solid tumors such as HCC. One of the advantages of CAR-T therapy for the treatment of HCC is that its efficacy is not dependent on antigen presentation with major histocompatibility complex (MHC) molecules, thus addressing tumor escape due to MHC downregulation [113]. Whereas OVs can be modified to express specific CAR antigens and successfully deliver them to the tumor surface, which can enhance the targeting of CAR-T cells [114]. The researchers found that the A56 antigen is upregulated in tumor cells infected by the oncolytic vaccinia virus (OVV), and they developed an A56-targeted CAR-T therapy. In a mouse model, the use of A56 CAR-T cells in combination with OVV and hydroxyurea significantly shrunk tumors and slowed progression, while reducing damage to normal cells and improving therapeutic efficacy against solid tumors [115]. In summary, OVs can be engineered to carry therapeutic transgenes that enhance the recognition and attack capabilities of CAR-T cells [116]. Combining oncolytic viruses’ tumor-killing capacity with the targeted immunity of CAR-T cells could overcome tumor resistance and greatly enhance treatment efficacy (Fig. 4).

### Oncolytic virotherapy combined with surgical therapy

Liver transplantation is currently the most effective treatment for liver cancer, not only eliminating the tumor but also providing a chance to cure the underlying liver disease. However, there is still a possibility of recurrence after liver transplantation, so many patients need to receive chemotherapy after liver transplantation to reduce the recurrence rate. Clinical trials have attempted to combine OVs with liver transplantation therapy, and the results showed that adjuvant ADV-TK therapy significantly delayed recurrence after liver transplantation in patients with advanced vascular infiltration HCC [117]. Meanwhile, it has been shown that VVΔTKΔN1L-IL12 (vaccinia virus armed with interleukin 12) significantly prolonged postoperative survival in a variety of cancer models and completely prevented tumor recurrence in a hamster model of head and neck cancer [118]. OVs acting in a localized spraying mode of action on tumors cause them to massively accumulate into cancerous tissues thereby generating a stronger expression of transgenes while inducing cellular and humoral immune responses, which can better inhibit tumor recurrence.

## The Adverse Events of the Oncolytic Virus

The safety of the OVs is generally acceptable, and the major adverse events identified to date include fever, malaise, chills, nausea, vomiting, lymphocytopenia, and leukopenia. For example, in the phase II clinical study of herpes simplex virus G47, the primary adverse event was fever, followed by vomiting, nausea, lymphopenia, and leukopenia [119]. In the phase II clinical trial of the JX-594 oncolytic virus, the primary side effects were fever and fatigue with lymphopenia [120]. Similarly, adverse reactions identified in a clinical trial of sequential treatment with JX-594 and sorafenib in patients with advanced HCC primarily included fever, chills, rash, hypotension, influenza-like illness, and injection-site pain, and patients in the combination therapy group were more likely to experience adverse events [97]. Whereas, we have found that corticosteroid administration rapidly reduces the number of these adverse effects associated with the immune response, such as fever and tumor swelling, and that this effect was sustained over a week without interfering with the development of anti-tumor immunity [121]. For grades 1 and 2 adverse reactions, symptomatic management and testing for changes in disease may allow the continuation of OV therapy. However, for grade 3 and higher adverse reactions (such as severe infections, hematopenia, neurotoxicity and so on that may lead to organ dysfunction), OV therapy should be discontinued immediately.

## Conclusion

Oncolytic virotherapy has emerged as a promising therapeutic modality in HCC treatment, offering selective oncolytic destruction and immune system activation, which minimizes side effects and enhances safety. Their utility extends beyond monotherapy, showing potential when combined with chemotherapy and immunotherapy. This combination strategy can overcome tumor heterogeneity and resistance associated with single-agent treatments by engaging multiple mechanisms of action. Despite their promise, oncolytic virotherapy encounters challenges such as the immunosuppressive tumor microenvironment, which may hinder viral replication and efficacy. Balancing the host immune response to prevent premature viral clearance while preserving the ability to kill cancer cells is crucial [122]. How to effectively deliver the oncolytic virus to the tumor site to play a role is also a major difficulty in the current practical application of OVs in HCC. Previously, OVs were attempted to administer systemically, but in this case, most of the viruses were neutralized by antibodies, complement, cytokines, and so on in the bloodstream or cleared by phagocytes before reaching the tumor site. A study of tumors from patients treated with OVs showed that even when patients received the highest dose (10^^^9 pfu), the amount of virus in the tumor remained low [123]. Currently, OVs are administered intratumorally, as this is currently the most effective mode of delivery. Although intratumoral injections are possible for solid tumors such as HCC, repeated intratumoral injections can induce a strong immune response to the virus and have limited efficacy against metastatic tumors [124]. Lastly, concerns regarding the safety profile, cost, storage, and complex administration protocols of OVs necessitate further investigation [125].

In conclusion, OVs exhibit significant potential in HCC therapy due to their targeted action, immune-activating properties, and combinatorial versatility, but they have both advantages and disadvantages. Advancements in genetic engineering may pave the way for the development of modified OVs, the construction of recombinant strains, and the elucidation of their molecular mechanisms, ultimately aiming to establish their long-term safety and efficacy. These efforts could lead to the development of more potent treatment regimens for patients with HCC.

## Data Availability

The datasets generated and/or analyzed during the current study are available from the corresponding author on reasonable request.
